# Comparison of the Toxicity of Pristine Graphene and Graphene Oxide, Using Four Biological Models

**DOI:** 10.3390/ma14154250

**Published:** 2021-07-29

**Authors:** Sławomir Jaworski, Barbara Strojny-Cieślak, Mateusz Wierzbicki, Marta Kutwin, Ewa Sawosz, Maciej Kamaszewski, Arkadiusz Matuszewski, Malwina Sosnowska, Jarosław Szczepaniak, Karolina Daniluk, Agata Lange, Michał Pruchniewski, Katarzyna Zawadzka, Maciej Łojkowski, Andre Chwalibog

**Affiliations:** 1 Department of Nanobiotechnology, Institute of Biology, Warsaw University of Life Sciences (WULS-SGGW), 02-787 Warsaw, Poland; barbara_strojny@sggw.edu.pl (B.S.-C.); mateusz_wierzbicki@sggw.edu.pl (M.W.); marta_kutwin@sggw.edu.pl (M.K.); ewa_sawosz@sggw.edu.pl (E.S.); malwina_sosnowska@sggw.edu.pl (M.S.); jaroslaw_szczepaniak@sggw.edu.pl (J.S.); karolina_daniluk@sggw.edu.pl (K.D.); agata_lange@sggw.edu.pl (A.L.); pruchniefski@gmail.com (M.P.); kasia.zaw7@gmail.com (K.Z.); 2 Department of Ichthyology and Biotechnology in Aquaculture, Institute of Animal Sciences, Warsaw University of Life Sciences (WULS-SGGW), 02-787 Warsaw, Poland; maciej_kamaszewski@sggw.edu.pl; 3 Department of Animal Breeding, Institute of Animal Sciences, Warsaw University of Life Sciences (WULS–SGGW), 02-787 Warsaw, Poland; arkadiusz_matuszewski@sggw.edu.pl; 4 Faculty of Materials Science and Engineering, Warsaw University of Technology, Wołoska 141, 02-507 Warsaw, Poland; 00183043@pw.edu.pl; 5 Centre for Advanced Materials and Technology CEZAMAT, Warsaw University of Technology, 02-822 Warsaw, Poland; 6 Department of Veterinary and Animal Sciences, University of Copenhagen, Groennegaardsvej 3, 1870 Frederiksberg, Denmark; ach@sund.ku.dk

**Keywords:** graphene, graphene oxide, toxicity, biological models

## Abstract

There are numerous applications of graphene in biomedicine and they can be classified into several main areas: delivery systems, sensors, tissue engineering and biological agents. The growing biomedical field of applications of graphene and its derivates raises questions regarding their toxicity. We will demonstrate an analysis of the toxicity of two forms of graphene using four various biological models: zebrafish (*Danio rerio*) embryo, duckweed (*Lemna minor*), human HS-5 cells and bacteria (*Staphylococcus aureus*). The toxicity of pristine graphene (PG) and graphene oxide (GO) was tested at concentrations of 5, 10, 20, 50 and 100 µg/mL. Higher toxicity was noted after administration of high doses of PG and GO in all tested biological models. Hydrophilic GO shows greater toxicity to biological models living in the entire volume of the culture medium (zebrafish, duckweed, *S. aureus*). PG showed the highest toxicity to adherent cells growing on the bottom of the culture plates—human HS-5 cells. The differences in toxicity between the tested graphene materials result from their physicochemical properties and the model used. Dose-dependent toxicity has been demonstrated with both forms of graphene.

## 1. Introduction

Graphene is a one-atom-thick sheet of carbon atoms arranged in a honeycomb-like pattern. Graphene family materials (GFM) including pristine graphene sheets (PG), few-layer graphene flakes, graphene oxide (GO), reduced graphene oxide (rGO) and graphene quantum dots offer various unique, versatile and tunable properties that can be used creatively for biomedical applications with a focus on cancer therapy, drug and gene delivery, bio-imaging and tissue engineering [1]. The growing biomedical field of applications of graphene-based materials raises questions regarding their toxicity. The interaction between dispersed GFM has been studied in vitro using human and animal cell cultures, such as HaCaT keratinocytes [2], human umbilical vein endothelial cells (HUVEC) [3], glioma cells [4,5,6], human multiple myeloma cells (RPMI-8226) [7,8], human mesenchymal stem cells (hMSCs) [9], red blood cells [10] and neuronal cells [11]. Many studies have shown that graphene toxicity depends on the size of the flakes. Akhavan et al. [9] showed significant cell destructions by 1.0 μg/mL rGO with average lateral dimensions (ALDs) of 11 ± 4 nm, while the rGO sheets with ALDs of 3.8 ± 0.4 μm could exhibit a significant cytotoxic effect only at a high concentration of 100 μg/mL after 1 h exposure time. Smaller flakes exhibit higher cellular uptake, internalization and higher cytotoxic effects [12]. The factor that also plays an important role is the number of oxygen functional groups that are attached to the surface. Liu et al. [13] presented that oxidized carbon nanomaterials—GO and oxidized single-wall carbon nanotubes (SWCNT-OH and SWCNT-COOH)—showed weaker cytotoxicity to the J774 cell line when compared to the corresponding non oxidized samples. Surface modifications of graphene, made by adding oxygen groups, improved their solubility and significantly reduced toxic interactions with cells [13]. The cytotoxic effects of graphene can be influenced by the size, shape, surface charge, surface area, lateral dimensions and surface chemistry [14,15]. The content of carboxyl groups on the edges and epoxy, hydroxyl and carbon radical groups on the surface contribute to toxic effects [16]. During the hydration, epoxy rings react with nucleophiles in aqueous solution, causing opening of the epoxy groups on GO surface and generating C-OH groups and carbon radicals. This enables the formation of a superoxide radical which, after joining the lipids of the cell membrane with the GO surface, leads to their oxidation and the formation of lipoperoxides [17]. Many GFM toxicity studies have also been performed using in vivo models: mice [18], rats [19] and fish [20]. Fu et al. [21] studied GO’s potential developmental toxicity when they entered the body of maternal mice and their offspring by oral exposure. The results showed that the increase in body weight, body length and tail length of the filial mice was significantly delayed compared with the group without GO. The anatomy and histology results revealed the retarded development of offspring in the high dosage group. However, in other studies, after intravenous administration, no pathological changes were observed in examined organs when mice were exposed to 1 mg kg^−1^ body weight of GO for 14 days. Moreover, GO showed long blood circulation time, good biocompatibility with red blood cells and low uptake in a reticuloendothelial system [22]. Similarly, GO nanoparticles had no toxic effects on blood parameters, growth and health of rats, suggesting the potential applicability of nanoparticles as drug carriers for local therapies [23,24].

The objective of this study was to evaluate the toxicity of PG and GO using various biological models: zebrafish (*Danio rerio*) embryo, duckweed (*Lemna minor*), human HS-5 cells and bacteria (*Staphylococcus aureus*). Each of the selected models is characterized by a varying degree of organization: from bacteria, through cell cultures, to entire plant and animal organisms. The presented experiments are preliminary studies that allow us to determine the toxicity of two forms of graphene and to observe the interactions between PG, GO and the biological structure. It is a necessary step to determine the predisposition of these materials for biomedical applications, such as antibacterial agents, active substances used in therapies or carriers of drugs. Moreover, the use of zebrafish (*Danio rerio*) embryo and duckweed (*Lemna minor*) as research models allows us to assess the ecological risk after the release of the tested nanomaterials into the environment.

## 2. Materials and Methods

### 2.1. Preparation and Characterization of PG and GO

Pristine graphene powder (PG) was purchased from SkySpring Nanomaterials (Houston, TX, USA), and graphene oxide (GO) was obtained from Warsaw University of Technology (Warsaw, Poland). The size and shape of the graphene flakes were evaluated using a JEM-1220 (JEOL, Tokyo, Japan) TEM at 80 KeV, with a Morada 11 megapixel camera (Olympus Soft Imaging Solutions, Münster, Germany). Zeta potential measurements were carried out using Zetasizer Nano S90 (Malvern Instruments Ltd., Malvern, UK). FT-IR measurements were performed using a Nicolet iS10 spectrometer (Thermo Fisher Scientific, USA), 200 mg of KBr powder (Sigma-Aldrich, Munich, Germany) and 0.5 mg of GO or PG was milled and pressed together under 10 Atm to form discs and then investigated in transmittance mode. The FT-IR spectrum was collected in the range 900–4000 cm^−1^.

### 2.2. Biological Models and Performed Analyses

The toxicity was tested using four biological models: zebrafish (*Danio rerio*) embryo and hatched larvae under FET test (Fish Embryo Acute Toxicity) conditions, the common duckweed (*Lemna minor*), bacteria *Staphylococcus aureus* and human HS-5 cells.

#### 2.2.1. Zebrafish (Danio rerio) Embryo

Fertilized zebrafish (*Danio rerio*) eggs were obtained from the Zebrafish Core Facility at the International Institute of Molecular and Cell Biology in Warsaw, Poland. For the toxicity test, water was prepared according to ISO 7346-3. The experiment conditions were described in detail by Nowakowska et al. [25]. An FET assay was conducted according to OECD guideline 236. The method was modified by using 6-well plates. Newly fertilized zebrafish AB/TL strain eggs (up to 2 h postfertilization) were transferred to media of different concentrations (5, 10, 20, 50 and 100 µg/mL) of PG and GO in 10 eggs per well. All experiments were performed in three replicates. The plates were incubated at 27 ± 0.5 °C for 96 h. Every 24 h of the experiment, from 24 h to 96 h, the number of living unhatched embryos, hatched larvae and dead and deformed individuals (larvae) in wells was counted using a binocular (Nikon SMZ 2000, Tokyo, Japan) with a camera (PixeLINK PL-A662). All dead embryos were removed to prevent contamination of the wells. After 96 h (hatching time), all dead embryos were counted along with the hatched fish embryos. The survival of embryos and larvae, the larval hatching rate at 96 h and the percentage of hatched larvae with pathological changes were calculated.

#### 2.2.2. Lemna Minor

*Lemna minor* was obtained from the Department of Ichthyobiology, Fisheries and Aquaculture Biotechnology, Warsaw, Poland University of Life Sciences. Maintenance and cultivation of fronds were carried out at 20 °C in 6-well plates with Steinberg growth medium [26] under static conditions. Continuous white fluorescent lighting was used to provide a light intensity from the range of 8500–9500 lx. There were 10 plants per well. Graphene was introduced to the medium at increasing concentrations (5, 10, 20, 50 and 100 μg/mL). After 96 h, frond damage, surface area, biomass and root length were analyzed using a Leica DM750 microscope coupled with a Leica ICC50 digital camera and cellSens microscope imaging software (Olympus Corporation, Warsaw, Poland). The area of the fronds was analyzed by autofluorescence readings using the Azure C400 system (Azure Biosystems, Dublin, Ireland).

#### 2.2.3. Staphylococcus Aureus

*Staphylococcus aureus* (ATCC 25923) was obtained from LGC Standards (Lomianki, Poland). The strain was stored as spore suspensions in 20% (*v*/*v*) glycerol at −20 °C. Prior to its use in experiments, the strain was defrosted and glycerol was removed by washing the bacterial cells with distilled water. The bacteria were grown on Mueller Hinton broth (BioMaxima, Lublin, Poland). The metabolic rate of bacterial cells was evaluated using a 2.3-Bis-(2-methoxy-4-nitro-5-sulfophenyl)-2H-tetrazolium-5-carboxyanilide salt (XTT)-based cell proliferation assay kit (Merck, Darmstadt, Germany) according to Jaworski et al. [4]. *S. aureus* cells were plated in 96-well plates (5 × 10^5^ cells per well). PG and GO were introduced to the cells at increasing concentrations (5, 10, 20, 50 and 100 μg/mL). After the addition of graphene, incubation was carried out for 24 h. In the next step, 50 μL of XTT solution was added to each well and incubated for an additional hour at 37 °C. The optical density (OD) of each well was recorded at 450 nm on a microplate reader. The interaction between graphene and bacteria was evaluated using TEM [27].

The assessment of intracellular reactive oxygen species level was conducted using a fluorometric intracellular ROS kit (Sigma, St Louis, MO, USA). Bacterial cells were plated in 96-well plates (5 × 10^5^ cells per well) with increasing concentrations of PG and GO. Afterwards, the Master Reaction Mix was added to the plate and fluorescence intensity was measured at 520 nm/605 nm with a microplate reader immediately and in 5-minute intervals. The test was performed in triplicate for each group.

#### 2.2.4. HS-5 Cells

HS-5 cells (bone marrow/stroma) were obtained from the American Type Culture Collection (Manassas, VA, USA) and maintained in Dulbecco’s modified Eagle’s culture medium containing 10% fetal bovine serum (Life Technologies, Houston, TX, USA), 1% penicillin and streptomycin (Life Technologies) at 37 °C in a humidified atmosphere of 5% CO_2_/95% air in a NuAire DH AutoFlow CO_2_ Air-Jacketed Incubator (Plymouth, MN, USA). The metabolic rate of HS-5 cells and intracellular ROS level was evaluated using the XTT and ROS assays, respectively (as above). The cell cycle was evaluated using flow cytometry. To assess the cell cycle, a method based on DNA staining with propidium iodide was used. HS-5 cells were seeded in the 6-well plates at 2 × 10^5^ cells per well and cultured until they reached 70–80% confluence. After that, the medium was removed and changed to the medium containing a 10% solution of the flakes of GO and PG at the final concentration of 20 and 50 µg/mL. A control sample consisted of cells cultured without the flakes. The UC San Diego Health Sciences protocol consisted of two steps: (1) fixation of cells and (2) staining with propidium iodide (PI, 500 µg/mL). The cells were washed twice with PBS, resuspended in PBS (1 mL) and fixed with nine volumes of 70% ethanol at 4 °C for 24 h at −20°C. After washing and centrifugation, each sample was resuspended in 0.5 mL of staining buffer (containing 10 mL of PBS buffer (*v*/*v*), 0.1% Triton X-100, 2 mg DNase-free RNase A and 0.4 mL propidium iodide at a concentration of 500 μg/mL). After incubating for 30 min in the dark, cells were analyzed by flow cytometry at a flow rate not exceeding 400 objects/s (FACSCalibur, Becton Dickinson, Franklin Lakes, NJ, USA). Twenty-four hours after exposure, the cell morphology was recorded using an optical microscope. 

## 3. Results and Discussion

### 3.1. Characteristics of PG and GO

**Figure 1:**
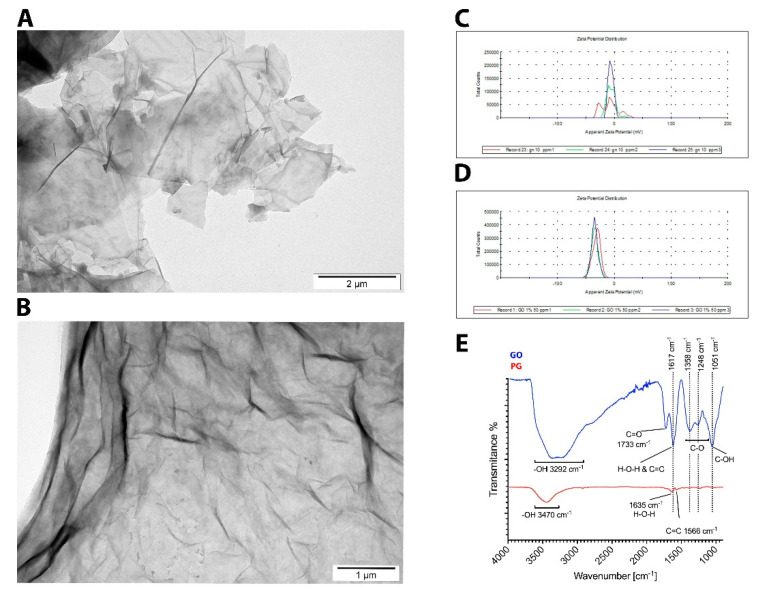
Physicochemical characteristics of the PG and GO: TEM images of agglomerated PG (**A**) and GO (**B**) flakes; zeta potential of PG (**C**) and GO (**D**); FT-IR (ATR, attenuated total reflectance) spectrum of PG and GO (**E**).

Figure 1 shows representative physicochemical characteristics of the PG and GO. Hydrophilic GO flakes formed a single layer. In contrast, most of the PG flakes were visible as a few layers and often created agglomerates. The shape of PG and GO was irregular with jagged edges. The surface diameter of the PG ranged from 350 nm to 6 μm. The GO flakes ranged from 100 nm to 2.5 μm. The mean zeta potential for the nanomaterials samples was −6.12 mV for PG, and −36.2 mV for GO. Pristine graphene usually does not have significant peaks that can be attributed as relevant to any functional groups [28]. However, in the PG sample, a wide band at 3470 cm^−1^ indicates −OH groups from adsorbed water. Two bands seen at 1635 cm^−1^ and 1566 cm^−1^ can be attributed to overlapping C=C aromatic ring vibrations and H–O–H scissoring vibrations originating from adsorbed water. In GO, the band at 1719 cm^−1^ is related to the C=O bond. A band at 1622 cm^−1^ can be attributed to C=C vibrations in aromatic ring, but it is more likely that this band originates from −OH vibrations from water molecules. At 1358 cm^−1^ and 1248 cm^−1^, the characteristic bands for GO can be seen, attributed to C–O epoxy or alkoxy groups, indicating incorporation of oxygen atoms inside the graphene structure. Strong peak at 1051 cm^−1^ can indicate the C–OH groups. 

### 3.2. Survival Rate and Morphology of Zebrafish Larvae

**Figure 2:**
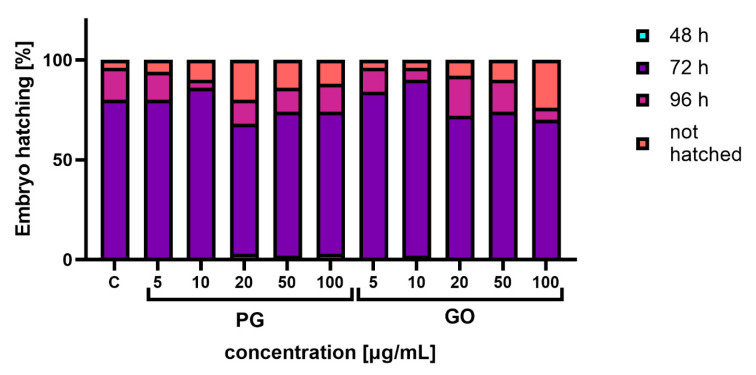
Percentage of larvae hatched after 48 hpf (hours after fertilization), 72 hpf and 96 hpf and percentage of non-hatched larvae on the last day of the experiment. Abbreviations: C—control group (untreated cells); PG—pristine graphene; GO—graphene oxide.

Analysis of the survival of embryos under FET test conditions showed that there was mass hatching after 72 h in the control group, and the number of unhatched embryos at the end of the FET test was about 4% (Figure 2). In the experimental groups exposed to PG, a higher percentage of unhatched embryos and individual larvae hatching after 48 h of incubation was observed. A similar trend was observed in the groups exposed to GO.

**Figure 3:**
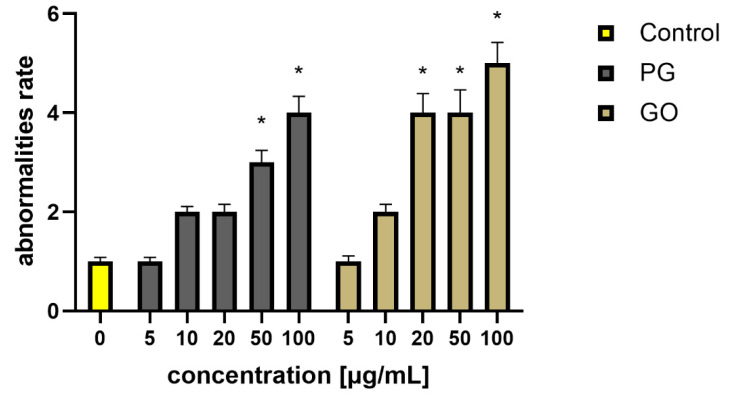
Percentage of abnormalities observed in the 96 h of the development of zebrafish embryos in the experimental groups. Values in the rows marked with an asterisk show statistically significant differences (*p* < 0.05) as compared to the control assay. Abbreviations: C—control group (untreated cells); PG—pristine graphene; GO—graphene oxide.

**Figure 4:**
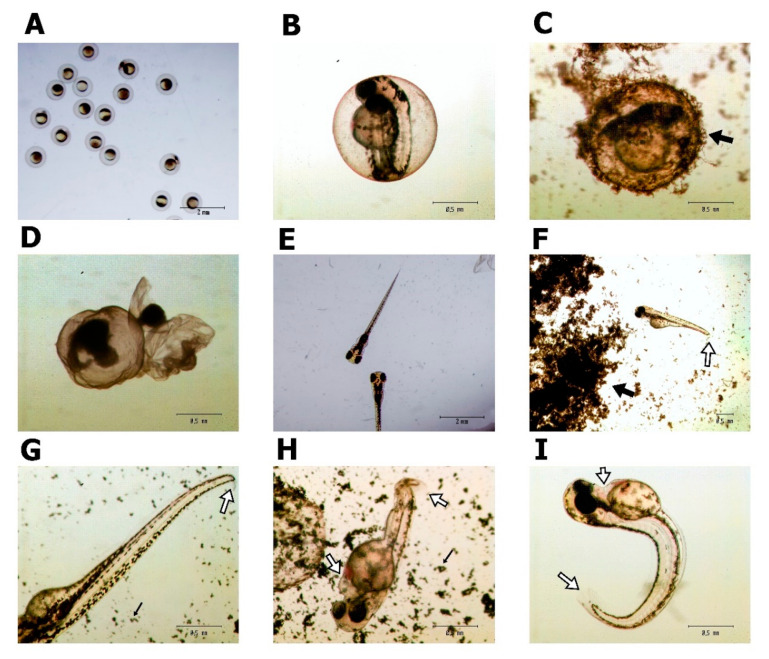
*Danio rerio* morphology after PG and GO treatment. (**A**,**B**) A zebrafish embryo 4 h after fertilization; (**C**) chorion covered with GO; (**D**) coagulated embryo; (**E**) properly developed embryo 96 h after fertilization, control group; (**F**–**I**) embryo deformities after PG (**F**,**H**) and GO (**G**,**I**) treatment. Black arrows indicate graphene agglomerates. White arrows indicate embryo deformities.

Analysis of the morphology of the larvae at 96 h after the FET test showed a significantly higher percentage of abnormalities in the groups PG50, PG100, GO20, GO50 and GO100 (Figure 3). Above all, changes such as malformation of the tail and pericardial oedema (Figure 4) were observed in the examined zebrafish. Moreover, in the groups with PG and GO at concentrations of 50 and 100 μg/mL, an accumulation of graphene flakes on the chorion surface was observed, which often led to death and coagulation of the embryo (Figure 4C,D).

In the control groups and groups exposed to the lowest concentrations of the tested nanomaterials (PG5 and GO5), the share of pathological changes was about 1% (Figure 3).

Many authors indicate that the presence of xenobiotics such as drugs, nanoparticles and others in aquatic ecosystems can cause teratogenic changes in fish. These changes include, among others, motor disorders, disorders of the structure of the axial skeleton, pericardial oedema, reduction in hatching or increased mortality [25,29]. However, data concerning the influence of carbon nanoparticles on zebrafish embryogenesis are limited so far. The obtained results indicate that both forms of graphene (PG and GO) influence the survival rate and hatching index of zebrafish embryos and larvae and the share of morphological abnormalities. Along with the increase in the concentration of the tested nanomaterials, a decrease in the number of hatched larvae after 72 h exposure and an increase in the share of non-hatched embryos were observed, as well as a significant increase in the pathological changes.

Similar effects were observed previously during the development of zebrafish exposed to reduced GO. Liu et al. [30] observed a reduction in the hatching rate in groups exposed to reduced GO, while an increase in the concentration of oxidized carbon nano-onions and oxidized carbon nano-horns reduced the survival of zebrafish embryos during exposure [31]. Moreover, Zhang et al. [32] found that even trace concentrations of GO can modify DNA and proteins and may lead to the increased generation of reactive oxygen species. These changes disturb homeostasis, including the expression of genes related to the development of the skeleton and heart [32], which may explain the observed pathological changes in the studied fish. Moreover, as found by Wang and Wang [29], one of the mechanisms of nanoparticle toxicity may be the agglomeration of nanoxenobiotics on the chorion surface, which may disrupt the oxygen and nutrient transport and may lead to embryo hypoxia and increased ROS production.

### 3.3. The Effect of PG and GO on Lemna Minor

**Figure 5:**
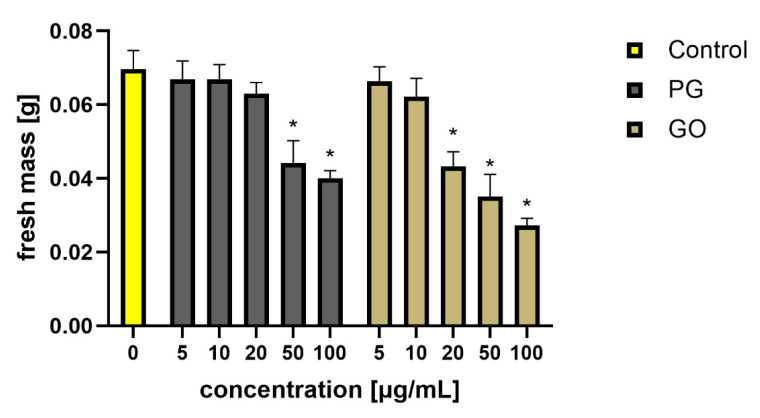
Average fresh mass of *Lemna minor* (10 plants per measurement) after PG and GO treatment. Values in the rows marked with an asterisk show statistically significant differences (*p* < 0.05) as compared to the control assay. Abbreviations: C—control group (untreated cells); PG—pristine graphene; GO—graphene oxide.

**Figure 6:**
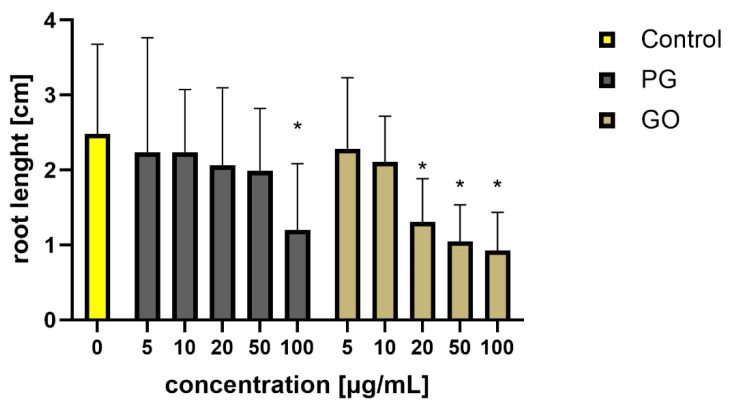
An average root length of *Lemna minor* treated with PG and GO. Values in the rows marked with an asterisk show statistically significant differences (*p* < 0.05) as compared to the control assay. Abbreviations: C—control group (untreated cells); PG—pristine graphene; GO—graphene oxide.

After 48 h of exposure to the nanomaterials, agglomeration of PG and GO flakes was noticed in the *L. minor* culture. Agglomeration was more visible in the groups exposed to PG. Some of the graphene flakes adhered to the roots. Fronds damage, surface area, biomass and root length were analyzed. There was a decrease in fresh mass in the groups treated with PG at concentrations of 50 and 100 μg/mL and GO at concentrations of 20 μg/mL and higher (Figure 5 and Figure 6).

**Figure 7:**
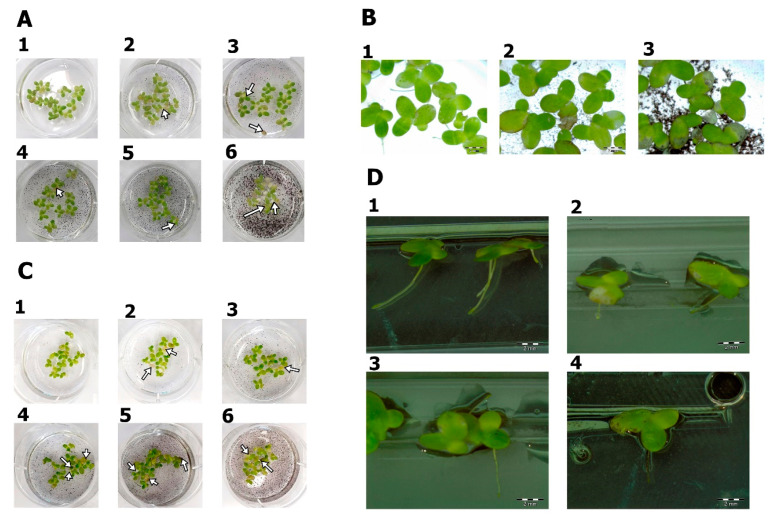
*Lemna minor* morphology after PG and GO treatment. (**A1**,**B1**,**C1**,**D1**) Control group; (**A2**–**A6**,**B2**,**D2**) after PG treatment; (**C2**–**C6**,**B3**,**D3**,**D4**) after GO treatment. White arrows indicate frond damage.

**Figure 8:**
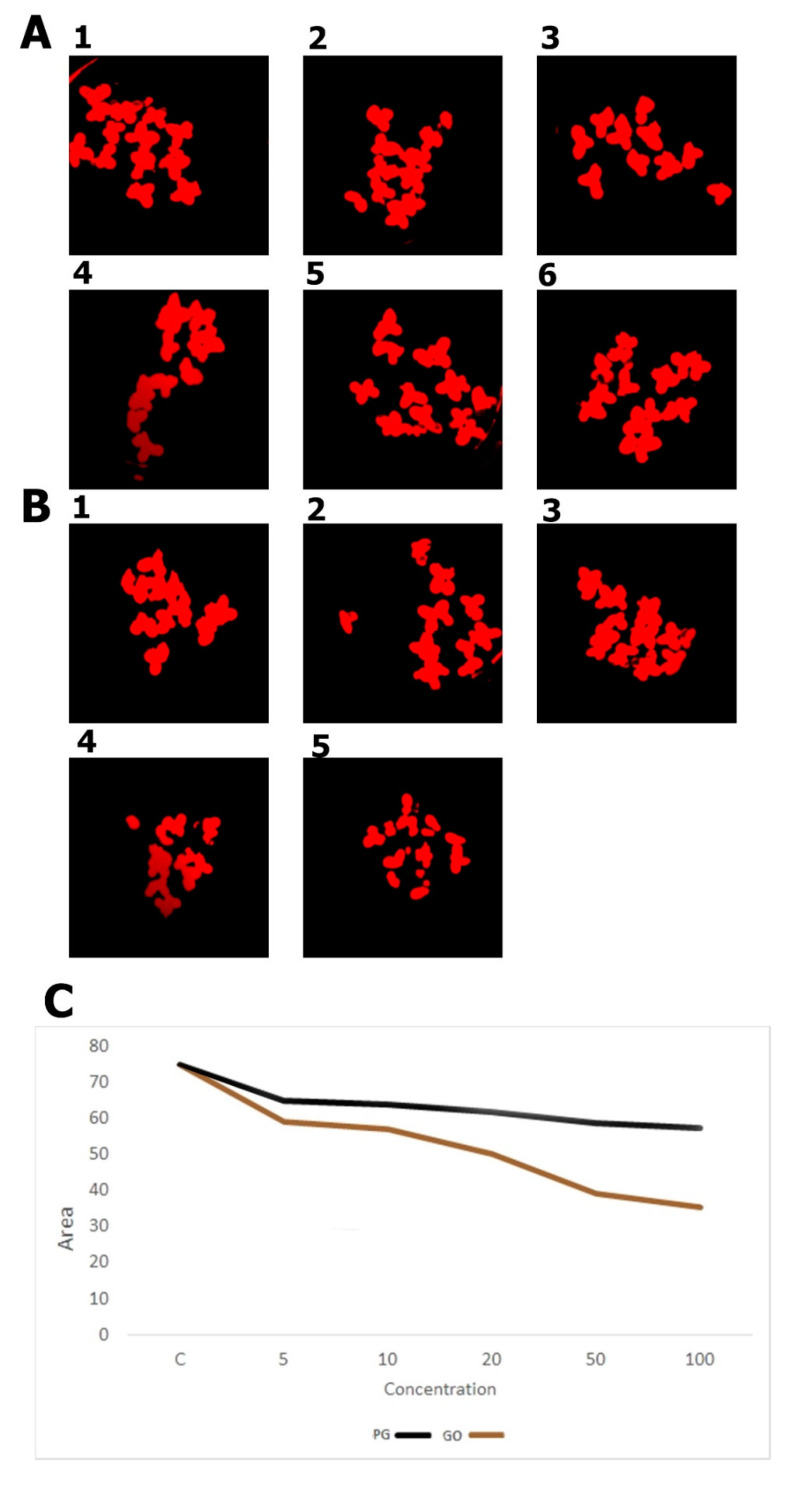
The area of fronds determined from the images after autofluorescence analysis. (**A1**) Control group; (**A2**) PG 5 μg/mL; (**A3**) PG 10 μg/mL; (**A4**) PG 20 μg/mL; (**A5**) PG 50 μg/mL; (**A6**) PG 100 μg/mL; (**B1**) GO 5 μg/mL; (**B2**) GO 10 μg/mL; (**B3**) GO 20 μg/mL; (**B4**) GO 50 μg/mL; (**B5**) GO 100 μg/mL; (**C**) calculated area of the fronds.

A smaller area of fronds, frond damage, white highlights on the surface and a decrease in root length were observed in PG groups at concentrations of 100 μg/mL and GO groups at concentrations of 20 μg/mL and higher (Figure 6, Figure 7 and Figure 8). The present results corroborate those of Castro et al. [33], who also showed a reduction in mass and leaf size after exposure to GO. It is difficult to discuss the results from PG as this is the first toxicity study of PG using this model. Graphene phytotoxicity was also evaluated by Begum [34]. A significant decrease in the weight and length of the roots of tomato, cabbage and red spinach exposed to graphene was shown. The observed influence depended on the concentration of graphene and the duration of the experiment. Differences in the weight of plants and the number of fronds after graphene treatment may result from graphene flakes sticking to the roots, which reduces the absorbent surface of the roots.

It has been demonstrated that the layered separative membranes based on GO flakes showed selective separation performance for ions, molecules, gases and organic solvents [35,36]. It is probable that the deposition of a thin layer of GO onto roots inhibits the capacity to adsorb compounds dissolved in water. These compounds deposit on the surface of the GO flakes, acting as a filter inhibiting the uptake of compounds and limiting plant growth.

### 3.4. The Antibacterial Effect of PG and GO

Graphene family materials (GFM) demonstrated a broad range of antimicrobial activity toward bacteria [37], fungi [38] and viruses [39]. These antibacterial activities are determined mainly by the direct physicochemical interaction between graphene and bacteria that causes a deadly disruption of cellular components—cell membranes and walls and nucleic acids [40]. So far, the antibacterial properties of graphene family materials have been demonstrated against a wide range of Gram-positive and Gram-negative bacteria.

**Figure 9:**
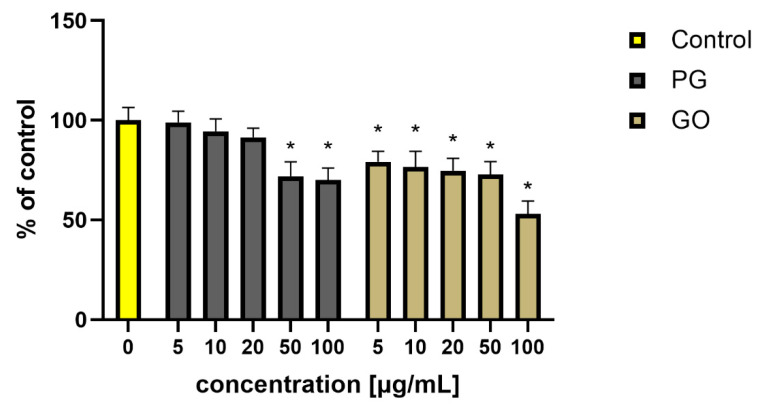
Viability of *S. aureus* (percent of the control) after PG and GO treatment. Values in the rows marked with an asterisk show statistically significant differences (*p* < 0.05) as compared to the control assay. Abbreviations: C—control group (untreated cells); PG—pristine graphene; GO—graphene oxide.

Experiments with *Staphylococcus aureus* showed a decrease in the viability of bacteria treated with PG in concentrations of 50 and 100 μg/mL and GO in all tested concentrations. The greatest inhibition of bacterial viability (53%) was noted in the group treated with GO at a concentration of 100 μg/mL (Figure 9). Additionally, in our previous studies, a more effective bactericidal effect was noted after incubation of *Listeria monocytogenes* and *Salmonella enterica* with GO flakes [27]. The high concentration (250 μg/mL) of PG, GO and rGO consistently inhibited the growth of both tested bacteria. At a lower concentration (25 μg/mL), only GO inhibited the growth of both bacteria totally, by 100% and 99.9% for *L. monocytogenes* and *S. enterica*, respectively.

**Figure 10:**
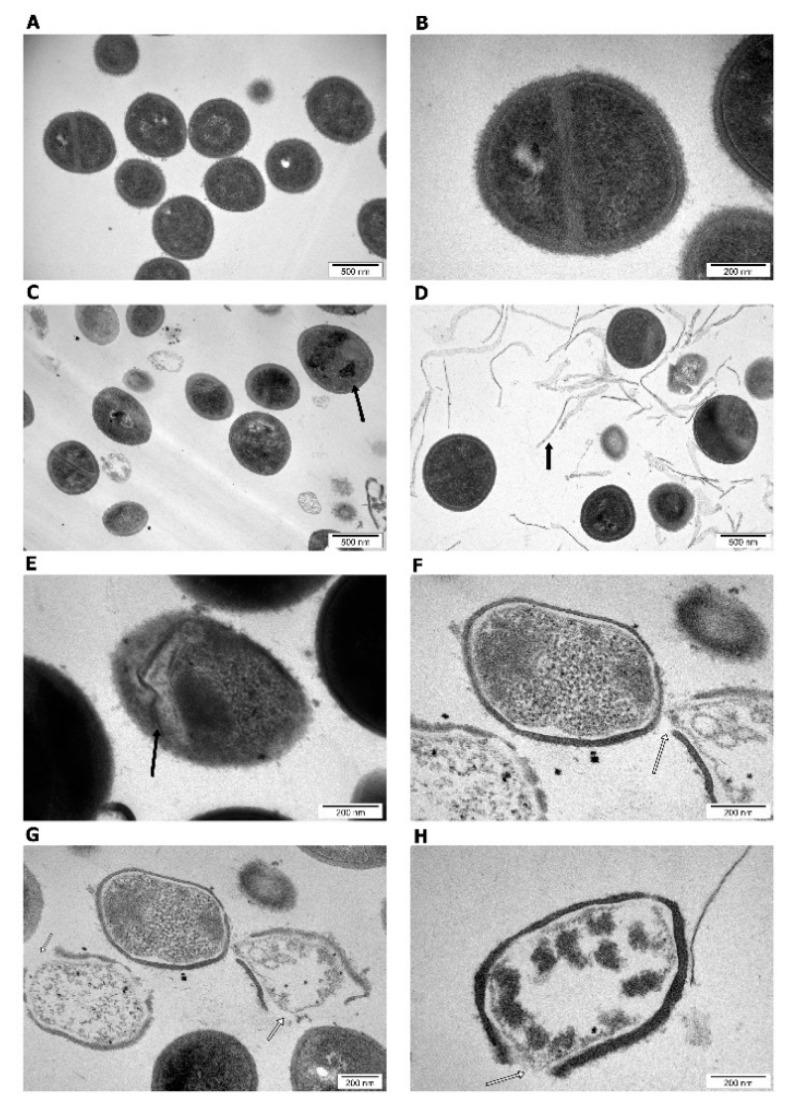
Morphology of *S. aureus* after PG and GO treatment. Transmission electron microscopy. (**A**,**B**) Control group; (**C**,**F**) PG-treated group; (**D**,**E**,**G**,**H**) GO-treated group. Black arrows indicate graphene agglomerates. White arrows indicate cell damage.

Direct contact with the bacterial outer surface may disrupt the cell wall and the membrane [41]. The sharp edges of PG and GO flakes might act as a “nano knife” and are particularly important for membrane stress since they enable efficient penetration of the phospholipid bilayers [42]. Analysis of the influence of graphene on the morphology and ultrastructure of the cell showed damage to the bacterial structures—cell wall and cell membrane in groups treated with PG and GO (Figure 10).

**Figure 11:**
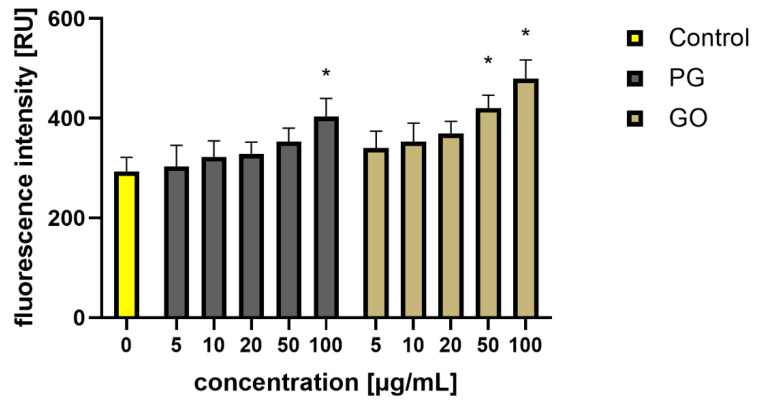
Analysis of the production of ROS in *S. aureus* cells after treatment with PG and GO. Values in the rows marked with an asterisk show statistically significant differences (*p* < 0.05) as compared to the control assay. Abbreviations: C—control group (untreated cells); PG—pristine graphene; GO—graphene oxide.

As a result, bacterial cells can suffer from disorders of membrane integrity that can prevent respiration, transport across the membrane and osmotic balance [17]. Ultimately, the damage is so great that the bacterial cells die (Figure 10). Adhesion of PG and GO flakes may interfere with respiration and energy transfer via the bacterial membrane, which causes production of reactive oxygen species (ROS). ROS react with membrane lipids and lead to the formation of lipid peroxides, which in turn oxidize and degrade other membrane components. To investigate ROS production as one of the key factors for cell death, the ROS levels were measured using intracellular ROS assay. The analysis showed an overproduction of ROS in the groups treated with PG at concentrations of 100 μg/mL and in the groups treated with GO at concentrations of 50 and 100 μg/mL (Figure 11).

Similar results were presented by Gurunathan et al. [43] in a study on *Pseudomonas aeruginosa*. Exposure to GO and rGO induced overproduction of the superoxide radical anion in comparison to the control group. Moreover, it was demonstrated before that the high number of oxygen-containing functional groups such as −OH and −COOH on the graphene surface enhances the production of ROS and increases the antimicrobial activity [44]. The structure of the external coatings of bacteria differs between bacterial species, which has an impact on the cell capacity to tolerate stress such as ROS-related lipid peroxidation and membrane disruption by sharp edges of graphene flakes. In comparison to Gram-negative bacteria, Gram-positive lacking the outer membrane are more sensitive to contact with sharp edges of graphene flakes [42,45].

### 3.5. The Influence of PG and GO on HS-5 Cells

**Figure 12:**
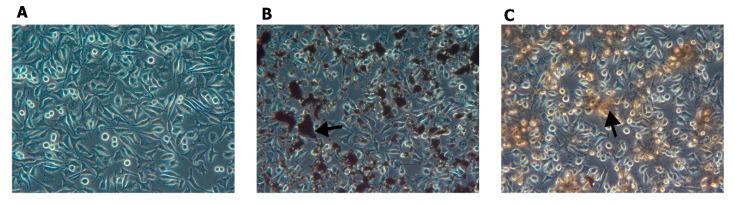
Morphology of HS-5 cells treated with PG and GO. (**A**) Control group; (**B**) PG group; (**C**) GO group. Black arrows indicate graphene and graphene oxide agglomerates.

Analysis of interactions between HS-5 cells and graphene showed high affinity of PG to the cells. PG flakes adhered to the cell body and formed agglomerates on the cell surface (Figure 12). GO adhered much less to the cells, and a significant part of the flakes was dispersed in the culture medium.

**Figure 13:**
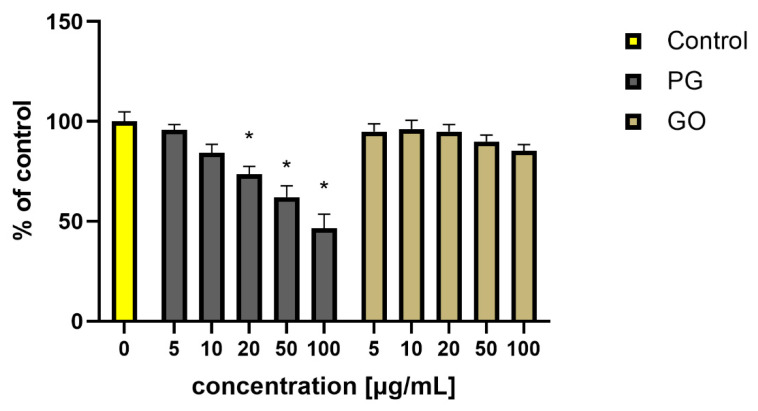
Viability of HS-5 cells (presented as a percentage of the control) treated with PG and GO. Values in the rows marked with an asterisk show statistically significant differences (*p* < 0.05) as compared to the control assay. Abbreviations: C—control group (untreated cells); PG—pristine graphene; GO—graphene oxide.

A similar phenomenon has been observed by other authors in studies using in vitro cell cultures [5,8,46]. To observe the behavior of PG and GO in the in vitro conditions, HS-5 cells were exposed to increasing concentrations of nanomaterials (5–100 μg/mL) and their cellular viability was determined using XTT assay after 24 h. The viability of HS-5 cells decreased after administration of PG at concentrations of 20–100 μg/mL. The lowest viability was observed in the group treated with PG at a concentration of 100 μg/mL (Figure 13).

**Figure 14:**
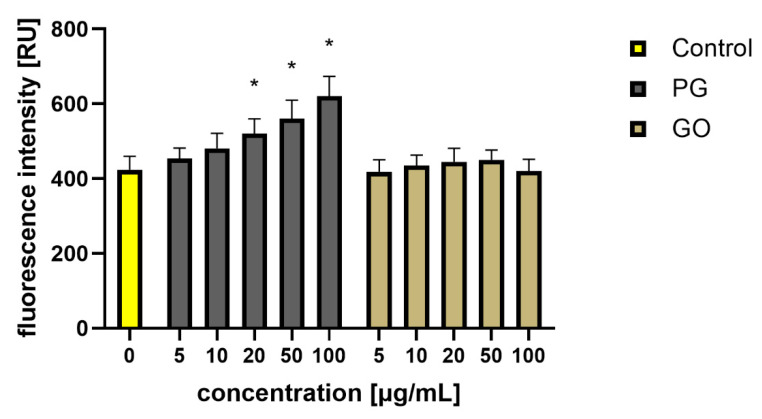
Analysis of the ROS production in HS-5 cells after treatment with PG and GO. Values in the rows marked with an asterisk show statistically significant differences (*p* < 0.05) as compared to the control assay. Abbreviations: C—control group (untreated cells); PG—pristine graphene; GO—graphene oxide.

Mittali et al. [47] also noted that unoxidized, thermally reduced GO induces higher viability reduction in comparison to GO. Some studies show that the gradually increasing total ROS content was exhibited in cells with GO or rGO treatment [48]. However, Gurunathan et al. [43] presented that the level of ROS production was significantly higher in rGO-treated cells than in GO-treated cells. In our investigation, an increase in the ROS level was observed in PG-treated groups at concentrations of 20 μg/mL and higher. Regardless of the concentration, GO did not reduce cell viability and did not increase ROS levels (Figure 14).

**Figure 15:**
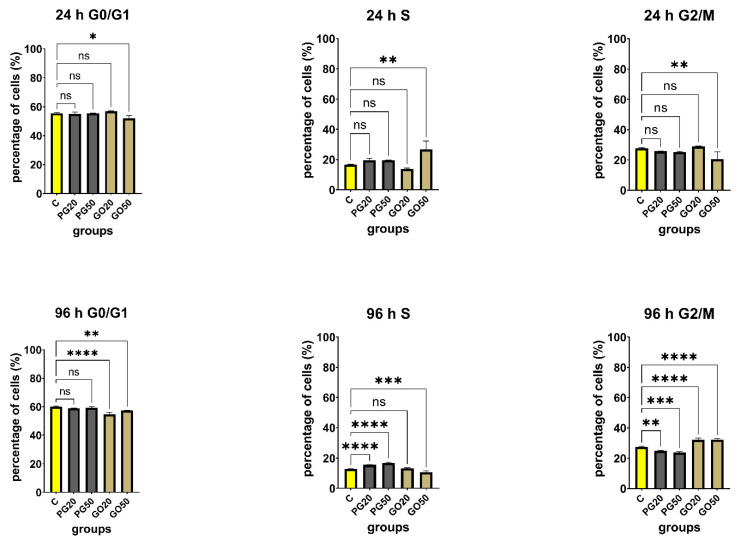
Cell cycle in Hs-5 cells untreated and treated with PG and GO flakes as evaluated by flow cytometry and propidium iodide (PI). The charts show the percentage of cells in individual phases of the cell cycle at all tested concentrations (20 µg/mL, 50 µg/mL), compared to the control groups (untreated). Values in the rows marked with an asterisk show statistically significant differences (*p* < 0.05) as compared to the control assay. Notes: G0—not dividing resting cells, cell cycle arrest; G1—preparation for division, cellular contents, excluding the chromosomes, are duplicated; S—phase synthesis DNA, each of the 46 chromosomes is duplicated by the cell; G2—the cell double checks the duplicated chromosomes for error, making any needed repairs; M—mitosis. Abbreviations: C—control group (untreated cells); PG—pristine graphene; GO—graphene oxide.

Previous studies have indicated that graphene family materials may lead to the arrest of the cell cycle at various phases. Hashemi et al. [49] showed that the accumulation of embryonic fibroblasts cells in the S phase was associated mainly with GO platelets. The results of this study showed that GO increased DNA synthesis through mechanisms such as ROS production and DNA damage and caused double-strand breaks in the DNA. Wang et al. [50] achieved similar effects, their results showing that functionalized GO significantly increased the S phase population together with decreased G1 population in HeLa, HEK293T, A549 and HepG2 cells. Cytometric analysis (Figure 15) showed significant differences in the percentage of cells in the individual phases of the cell cycle. After 24 h treatment with GO at 50 µg/mL, a significant (9.865%; *p*-value < 0.05) increase in the percentage of cells in the S phase of the cycle was observed in comparison with control cells, which may indicate the cell cycle arrest in this phase. A significant decrease in the percentage of cells in the G0/G1 (3.59%; *p*-value < 0.05) and G2/M (7.388%; *p*-value < 0.05) phase was observed in the same group. Ninety-six hours after treatment, statistically significant differences were observed for both study groups, both in GO and GN. In the G0/G1 phase, the most statistically significant result was seen in the GO group at a concentration of 20 µg/mL, where there was a decrease (5.3%; *p*-value < 0.05). In the S phase, a statistically significant increase was observed in the PG 20 µg/mL (2.725%; *p*-value < 0.05) and 50 µg/mL (4.065%; *p*-value < 0.05) groups. In the G2/M phase, statistically significant results were observed in each of the studied groups, with a distinct increase in the GO 20 µg/mL (4.79%; *p*-value < 0.05) and 50 µg/mL (4.805%; *p*-value < 0.05) groups. In the study groups with PG, a decrease was observed in this phase at 20 µg/mL (2.415%; *p*-value < 0.05) and 20 µg/mL (3.7%; *p*-value < 0.05). The results primarily show that depending on whether we are dealing with GO or PG flakes, the cell cycle is inhibited in other phases of the cycle. After 96 h of the treatment, GO flakes arrested the cell cycle in the G2/M phase, while PG flakes did so in the S phase.

## 4. Conclusions

The differences in toxicity between the tested graphene materials result from their physicochemical properties and the model used. Hydrophilic GO shows greater toxicity to biological models living in the entire volume of the culture medium (zebrafish, duckweed, *S. aureus*). Good distribution in the medium increases the possibility of contact with the cell/organism living in the fluid medium. PG is characterized by much lower stability in aqueous solutions; it quickly aggregates and sinks to the bottom of the culture plates. It also limits the contact with organisms living in planktonic cultures. PG showed the highest toxicity to adherent cells growing on the bottom of the culture plates—HS-5 cells.

## Data Availability

The data presented in this study are available on reasonable request from the corresponding author.
